# An* In Vitro* Comparison of PMMA and Calcium Sulfate as Carriers for the Local Delivery of Gallium(III) Nitrate to Staphylococcal Infected Surgical Sites

**DOI:** 10.1155/2016/7078989

**Published:** 2016-01-17

**Authors:** Rebecca A. Garcia, David J. Tennent, David Chang, Joseph C. Wenke, Carlos J. Sanchez

**Affiliations:** Extremity Trauma and Regenerative Medicine, United States Army Institute of Surgical Research, JBSA Fort Sam, Houston, TX, USA

## Abstract

Antibiotic-loaded bone cements, including poly(methyl methacrylate) (PMMA) and calcium sulfate (CaSO_4_), are often used for treatment of orthopaedic infections involving* Staphylococcus* spp., although the effectiveness of this treatment modality may be limited due to the emergence of antimicrobial resistance and/or the development of biofilms within surgical sites. Gallium(III) is an iron analog capable of inhibiting essential iron-dependent pathways, exerting broad antimicrobial activity against multiple microorganisms, including* Staphylococcus* spp. Herein, we evaluated PMMA and CaSO_4_ as carriers for delivery of gallium(III) nitrate (Ga(NO_3_)_3_) to infected surgical sites by assessing the release kinetics subsequent to incorporation and antimicrobial activity against* S. aureus* and* S. epidermidis*. PMMA and to a lesser extent CaSO_4_ were observed to be compatible as carriers for Ga(NO_3_)_3_, eluting concentrations with antimicrobial activity against planktonic bacteria, inhibiting bacterial growth, and preventing bacterial colonization of beads, and effective against established bacterial biofilms of* S. aureus* and* S. epidermidis*. Collectively, our* in vitro* results indicate that PMMA is a more suitable carrier compared to CaSO_4_ for delivery of Ga(NO_3_)_3_; moreover they provide evidence for the potential use of Ga(NO_3_)_3_ with PMMA as a strategy for the prevention and/or treatment for orthopaedic infections.

## 1. Introduction

Orthopaedic related postoperative infections are a serious complication contributing to the increased overall healthcare associated costs as well as patient associated morbidity [1–4]. For traumatic open lower extremity fractures, infectious complications occur in up to as many as 64% of patients and is a significant factor contributing to increased rates of surgical revisions, time to osseous union, and extremity amputation [5–8]. While the vast majority of orthopaedic related infections involve Gram positive bacteria, including methicillin resistant* Staphylococcus aureus *(MRSA) and* Staphylococcus epidermidis* [9], infections due to Gram negative bacteria have also been described in particular for traumatic orthopaedic injuries [6, 10]. Of note, the majority of bacteria responsible for these infections display resistance to a number of the commonly used antibiotics for treatment, further complicating the clinical management [6, 10–12].

The current standard of care for the majority of orthopaedic related wound infections is a combination of surgical management and systemic antibiotics, often with the addition of local antimicrobial therapy [2, 3, 13–15]. Controlled release of local antibiotics to infected surgical sites is typically achieved using nonabsorbable or absorbable carriers such as poly(methyl methacrylate) (PMMA) and calcium sulfate, respectively [13, 16, 17]. The most common antimicrobials loaded in bone cements include broad spectrum aminoglycosides, such as gentamicin and tobramycin, and/or the glycopeptide, vancomycin. While PMMA beads are traditionally used for local antibiotic delivery, the variability of antimicrobial elution from these materials, the limited compatibility due to the exothermic polymerization of the material, and the requirement of a secondary surgery for removal have led to more frequent clinical use of absorbable materials including calcium sulfate [1, 18, 19]. Importantly, despite the success associated with use of this treatment modality, the emergence of antimicrobial resistance amongst organisms as well as biofilm formation has been reported to reduce the effectiveness of this intervention [4, 20], highlighting the need for use of alternative agents to address this growing clinical challenge.

The acquisition of ferric iron(III) (Fe(III)) from the surrounding environment is critical to normal bacterial physiology and virulence. However, ferric iron bioavailability in soft tissues is normally kept at extremely low levels (<10^−18 ^M) as a part of an immune defense against invading pathogens [21]. In response to the low availability of iron, bacteria have evolved numerous strategies enabling iron acquisition [22, 23]. Given this pivotal role of iron, there have been numerous studies evaluating the potential of iron modulation as an alternative method of antimicrobial therapy [24–27]. In particular, the nonreducible iron analog, gallium(III), commonly used as the salt gallium(III) nitrate ((GaNO_3_)_3_) and the active component of the previously FDA approved drugs used for treating bone loss disorders such as Paget's and hypercalcemia [28–30], has been shown to have broad antimicrobial activity against both Gram negative and Gram positive species, including *Staphylococcus* spp. [25, 26, 31–33]. Given the chemical similarity of gallium(III) to ferric iron, gallium can effectively compete with, bind, and inhibit the activity of iron-dependent enzymes exerting strong antimicrobial activity [24, 25]. As iron has been shown to be essential to bacterial growth and virulence, and moreover to modulate biofilm formation* in vitro*, use of Ga(NO_3_)_3_ may represent an effective strategy for the prevention and treatment of infections. While there have been studies demonstrating the successes of intravenous use of Ga(NO_3_)_3_ for the treatment of systemic bacterial infections [34, 35], to our knowledge there are no studies to date that have evaluated the use of Ga(NO_3_)_3_ for treatment of orthopaedic related infections.

As antibiotic-loaded PMMA and calcium sulfate have been traditionally used as preventative and treatment strategies for orthopaedic related infections, the purpose of this study was to determine whether PMMA and/or CaSO_4_ could be used as carriers for local delivery to infected surgical sites by assessing the release kinetics and evaluating antimicrobial activity against planktonic and biofilm derived staphylococci* in vitro*.

## 2. Materials and Methods

### 2.1. Reagents

Gallium(III) nitrate ((GaNO_3_)_3_) was purchased from Sigma-Aldrich (St. Louis, MO) and prepared for use in the experimental assays according to the manufacturer's recommendations.

### 2.2. Bacterial Strains and Culture Conditions

In this study commercially available strains from the American Type Culture Collection (ATCC, Manassas, VA, USA), including* Staphylococcus aureus* ATCC 29213 and* Staphylococcus epidermidis* ATCC 12228 were used. Bacterial strains were cultured on Mueller-Hinton Agar Plates (Remel, Lenexa, KS, USA) or in Cation-adjusted Mueller-Hinton broth (MHB II) at 37°C.

### 2.3. Preparation of Poly(methyl methacrylate) (PMMA) and Calcium Sulfate Beads

PMMA beads loaded with 2.4%, 4.7%, 9.09%, and 13% Ga(NO_3_)_3_ were made by combining 40 g PALACOS R Radiopaque bone cement powder (Zimmer Orthopaedic Surgical Products, Dover, OH, USA) with 0.983 g, 1.98 g, 3.9 g, and 5.8 g of Ga(NO_3_)_3_ powder, respectively. Methyl methacrylate monomer (20 mL) was added to the powder, mixed thoroughly, and spread across a 3 mm mold, creating beads weighing approximately 20 mg each. For the preparation of Ga(NO_3_)_3_ loaded calcium sulfate beads at similar concentrations, 10 cc bone cement (Osteoset Resorbable Mini-Bead Kit, Wright Medical Technology, Inc., Netherlands) was mixed with 0.246 g, 0.492 g, 0.99 g, and 1.46 g, respectively. CaSO_4_ beads were casted using the 3 mm molds as described as above. A qualitative assessment on the effect of Ga(NO_3_)_3_ loading on the curing time of PMMA and CaSO_4_ beads was performed by testing the firmness of the materials over time relative to beads loaded with a clinically relevant amount of the glycopeptide, vancomycin (2.4% w/w) [1, 36]. This comparison was primarily performed to demonstrate the effect of Ga(NO_3_)_3_ to increase curing time which could limit its potential clinical utility, given that antibiotic loaded beads are typically prepared during surgical procedures.

### 2.4. Ga(NO_3_)_3_ Release Kinetics

For collection of eluents from the Ga(NO_3_)_3_ loaded PMMA and calcium sulfate, beads (three/group) were placed into 2 mL of PBS and incubated at 37°C as previously described [18, 37]. Eluents were removed daily, collected, and tubes containing beads were replenished with fresh PBS daily for up to 7 days. The collected eluents were stored at −80°C until use.

Quantification of gallium (Ga) was accomplished using Inductively Coupled Plasma Mass Spectrometry (ICP-MS) of acid digested samples. Briefly, 150 *μ*L BDH Aristar Plus Nitric Acid (70%, VWR Scientific, Radnor, PA, USA) was added to metal-free 15 mL conical tubes, followed by 150 *μ*L sample. Samples were then heated at 80°C for 4 hours followed by addition of ultrapure H_2_O (18.2 MΩ·cm) and multielement internal standard containing Bi, Ho, In, 6Li, Sc, Tb, and Y (CLISS-1, Spex CertiPrep, Metuchen, NJ, USA) to produce a final solution of 3% nitric acid (v/v) and 1 ng/mL internal standard in a total sample volume of 5 mL. Individual Ga elemental standards were prepared by diluting a 1000 *μ*g/mL of certified Ga standard (Inorganic Ventures, Christiansburg, VA, USA) to 10 *μ*g/mL Ga. Ga standards were then made via 1/2 serial dilutions to obtain 9 elemental standards and a blank. All standards contained 3% nitric acid (v/v) and 1 ng/mL internal standard up to a total sample volume of 5 mL. ICP-MS was performed on a computer-controlled (Qtegra software v. 2.4) Thermo iCap Qc ICP-MS (Thermo Fisher Scientific, Waltham, MA, USA) operating in standard mode and equipped with a CETAC 260 Autosampler (Omaha, NE, USA). Each sample was acquired using a 35 sec uptake and 90 sec washout time (rinse was 3% Aristar Plus HNO_3_ (v/v)), 1 survey run (3 sweeps, 10 ms dwell time), and 3 main (peak jumping) runs (100 sweeps, 100 ms dwell time). The isotopes selected for analysis were 69,71Ga, with 89Y and 115In chosen as internal standards for data interpolation. Instrument performance is optimized daily through autotuning followed by verification. Absolute values of gallium [*μ*M] as well as the cumulative release over time, as expressed as percentage released from the total original loaded amount into PMMA or CaSO_4_, were plotted.

### 2.5. Antimicrobial Activity Ga(NO_3_)_3_ against Planktonic Bacteria

The inhibitory concentration of Ga(NO_3_)_3_ was determined using a modified version of the broth microdilution assay in 96-well round bottom plates as previously described [18, 20, 34, 35]. In brief, bacteria were grown in MHB II broth to an optical density (600 nm) of 0.1 (~10^8^ CFU/mL), washed, and resuspended in diluted MHB II (0.3 g/L) broth to a final bacterial concentration of 10^6^ CFU/mL. One hundred microliters was then transferred to individual wells of a round bottom plate (~10^5^ CFU/well), containing 100 *μ*L of Ga(NO_3_)_3_ at increasing concentrations, 0.25–512 *μ*M, diluted in MHB II (0.3 g/L) broth at a 2x concentration. Bacteria were incubated overnight at 37°C under static conditions, and following overnight incubation, the optical densities (*A*_600 nm_) were measured.

### 2.6. Use of Ga(NO_3_)_3_ Loaded PMMA and CaSO_4_ Beads to Inhibit Bacterial Growth in Broth

To evaluate the antimicrobial ability of Ga(NO_3_)_3_ loaded beads at the various concentrations against planktonic bacteria in broth cultures, beads (3/group) were added to sterile 15 mL conical tubes containing 5 mL of a bacterial culture adjusted to 10^6^ CFU/mL in MHB II (0.3 g/L) broth. Cultures containing the Ga(NO_3_)_3_ beads were coincubated at 37°C with agitation for up to 7 days, with cultures removed and exchanged every 24 hours. At days 1, 3, and 7, 100 *μ*L was removed and bacterial viability within cultures was determined by plating serial dilutions onto MHB agar plates. As a control group for the experiment, unloaded (0.0%) PMMA and CaSO_4_ beads were used. Data was represented as the log reduction relative to a bacterial culture grown in MHB II (0.3 g/L) broth without PMMA or CaSO_4_ beads.

### 2.7. Bacterial Colonization of Ga(NO_3_)_3_ Loaded PMMA and CaSO_4_ Beads

To evaluate the effect of Ga(NO_3_)_3_ loading on the bacterial colonization of PMMA and CaSO_4_ beads simultaneously with elution, Ga(NO_3_)_3_ loaded and unloaded beads (6/well) were placed into 6-well plates and cultured up to 7 days as previously described [20]. Briefly, 4 mL of MHB II broth, 0.3 g/L, containing 10^6^ CFU/mL of bacteria was added to the individual wells containing the PMMA or CaSO_4_ beads containing the increasing concentrations of Ga(NO_3_)_3_. Every 24 hours, broth cultures were removed and replaced with fresh bacterial cultures exposing the beads to continuous bacterial challenge. At days 1, 3, and 7, beads were removed from plates, washed with sterile 1x PBS, and placed into individual wells of a 96-well plate, and the plates containing beads were sonicated to remove attached bacteria. The number of viable bacteria removed from beads was determined by plating serial dilutions onto MHB agar plates as previously described [38].

### 2.8. Activity of Ga(NO_3_)_3_ Loaded Beads on Established Bacterial Biofilms

Biofilms were developed and evaluated for susceptibility to Ga(NO_3_)_3_ using the minimum biofilm eradication concentration (MBEC) P&G plates (Innovotech, Alberta, Canada) as previously described with some minor modifications [38–40]. In brief, 180 *μ*L of bacteria diluted to 10^6^ CFU/mL was added to individual wells of the MBEC plates and incubated for 48 hours at 37°C with shaking at 150 rpm (VWR, Radnor, PA, USA). Following incubation, the plate tops containing the pegs with established biofilms were rinsed in sterile 1x PBS, placed in a challenge plate containing either Ga(NO_3_)_3_, 0.25–512 *μ*M, or Ga(NO_3_)_3_ loaded beads (2.4–13%) in MHB II (0.3 g/L) broth and incubated for an additional 24 hours. After treatment, pegs were then rinsed and sonicated for 15 minutes at 40 kHz (Branson Ultrasonics Corp., Danbury, CT, USA) into a 96-well plate containing PBS. Bacterial viability was determined by plating serial dilutions onto MHB agar plates.

### 2.9. Statistical Analysis

Where appropriate, statistical analysis was performed using an unpaired Student *t*-test or a one-way ANOVA with Dunnett's post hoc evaluation for comparison of the control group between multiple treatment groups. Values of *p* < 0.05 were considered to be statistically significant. All experimental assays were performed in triplicate.

## 3. Results 

### 3.1. Activity of Ga(NO_3_)_3_ on Planktonic Culture and Established Biofilms of* S. aureus* and* S. epidermidis*

**Figure 1:**
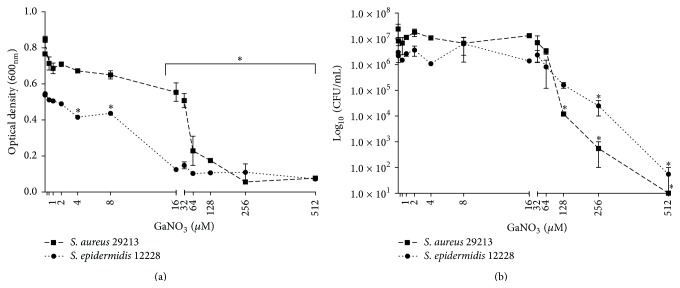
*In vitro* antimicrobial activities of Ga(NO_3_)_3_. Activity of Ga(NO_3_)_3_, 0.5–512 *μ*M, against planktonic bacteria (a) and biofilms (b) of* Staphylococcus epidermidis* ATCC 12228 and* Staphylococcus aureus *ATCC 29213 following overnight exposure to increasing concentrations, in MHB II (0.3 g/L) broth in 96-well plates. Data is representative of mean ± SD of three independent experiments. Statistical analysis was performed using Student's *t*-test; *∗* indicates *p* < 0.05 relative to the untreated control group; Student's *t*-test.

Initial testing of the effect of Ga(NO_3_)_3_ on planktonic growth of* S. aureus* ATCC 29213 and* S. epidermidis* ATCC 12228 was performed using a modified version of the broth microdilution assay in iron deplete media to assess antimicrobial activity and ensure bacterial susceptibility of the strains used in the study. Antimicrobial activity of Ga(NO_3_)_3_ on planktonic, that is, culture grown, bacteria was observed to be both strain- and concentration-dependent with significant decreases in bacterial growth at concentrations ≥16 *μ*M and ≥4 *μ*M for* S. aureus* and* S. epidermidis*, respectively (Figure 1(a)). Notably, concentrations of Ga(NO_3_)_3_ ≥64 *μ*M were observed to completely inhibit bacterial growth of both strains tested. In addition to the activity on planktonic bacteria, Ga(NO_3_)_3_ was also observed to have antimicrobial activity against biofilms of* S. aureus *and* S. epidermidis*, albeit at much higher concentrations, ≥128 *μ*M and ≥256 *μ*M, respectively, compared to their planktonic counterparts (Figure 1(b)).

### 3.2. *In Vitro* Release of Ga(NO_3_)_3_  from PMMA and CaSO_4_

To evaluate the potential use of PMMA and CaSO_4_ as carriers for Ga(NO_3_)_3_ we evaluated the effect of loading various concentrations on the curing time of these materials; moreover we characterized the release kinetics of gallium(III) over time. Incorporation of Ga(NO_3_)_3_ into PMMA at concentrations ≥9.09% (w/w) extended the time for the curing of PMMA roughly up to 1 hour compared to the approximately ~15 minutes required for curing of vancomycin loaded PMMA (2.4% w/w). Of note, while the PMMA beads were not completely cured, even after 30 min, the material was workable and maintained structural integrity with handling. In contrast to PMMA, incorporation of Ga(NO_3_)_3_ into CaSO_4_ up to the 13% (w/w) was not observed to have any impact on the curing time, relative to beads loaded with vancomycin.

**Figure 2:**
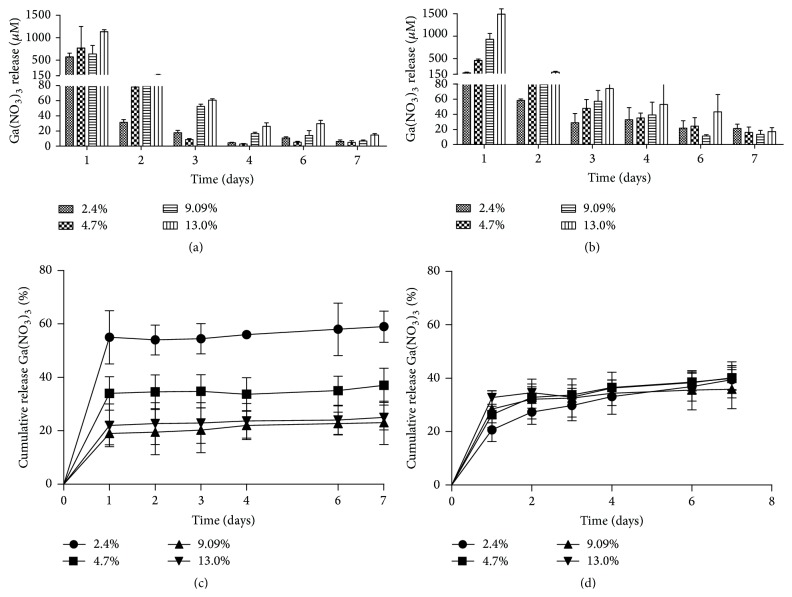
Characterization of Ga(NO_3_)_3 _release kinetics from PMMA and CaSO_4_ beads. Absolute release of Ga(NO_3_)_3 _from PMMA (a) and CaSO_4_ (b) beads, loaded at 2.4, 4.7, 9.09, and 13% (w/w), over 7 days performed in PBS is shown. The percent cumulative release of the total amount of Ga(NO_3_)_3_ (by weight) loaded into PMMA (c) and CaSO_4_ (d) beads is shown. Values are reported as the mean ± SD of *n* = 3 samples.

Release of Ga(NO_3_)_3_ from both PMMA and CaSO_4_ was characterized by a rapid initial release followed by slower sustained release. Ga(NO_3_)_3_ release from PMMA had a large initial burst, releasing 55%, 34%, 19%, and 22% of the total amount loaded within the first day and reaching mean concentrations of 470 ± 9, 592 ± 11, 636 ± 10, and 1149 ± 11 *μ*M, for the 2.4%, 4.7%, 9.09%, and 13% (w/w), respectively (Figures 2(a) and 2(c)). After this initial burst, elution of Ga(NO_3_)_3_ was much lower and sustained for up to 7 days releasing 59%, 37%, 23%, and 25% and reaching mean levels of 6 ± 5, 5 ± 3, 7 ± 4, and 14 ± 5 *μ*M, for 2.4%, 4.7%, 9.09%, and 13% (w/w), respectively. Similarly, Ga(NO_3_)_3_ release from CaSO_4_ also had a large initial burst releasing 21%, 26%, 28%, and 28% within the first day and reaching mean concentrations of 178 ± 13, 458 ± 11, 929 ± 13, and 1488 ± 15 *μ*M, for 2.4%, 4.7%, 9.09%, and 13% (w/w), respectively (Figures 2(b) and 2(d)). Ga(NO_3_)_3_ release from CaSO_4_ was detected up to the 7 days evaluated, releasing 39%, 39%, 35%, and 36%, and reaching mean levels of 21 ± 4, 16 ± 3, 13 ± 3, and 17 ± 8 *μ*M, for 2.4%, 4.7%, 9.09%, and 13% (w/w), respectively.

### 3.3. Inhibitory Activity of Ga(NO_3_)_3_ Loaded PMMA and CaSO_4_ Beads

**Figure 3:**
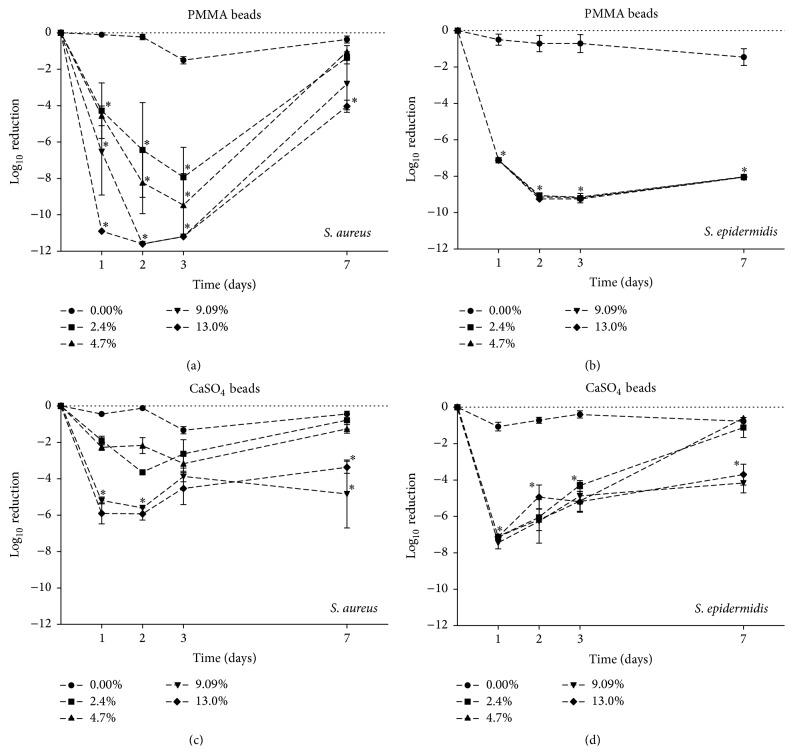
Activity of Ga(NO_3_)_3_ loaded PMMA and CaSO_4_ beads on planktonic bacteria. Antimicrobial activity of PMMA (a-b) and CaSO_4_ (c-d) loaded Ga(NO_3_)_3_ beads on bacterial cultures of* S. aureus* ATCC 12913 and* S. epidermidis* ATCC 12228 over time. Data are expressed as log reduction relative to empty (0.0%) PMMA/CaSO_4_ beads. Values are reported as the mean ± SD. Statistical analysis was performed using an analysis of variance (ANOVA) with Dunnett's post hoc test; *∗* indicates *p* < 0.05 relative to control.

To evaluate the antimicrobial activity against* S. aureus* and* S. epidermidis*, bacterial cultures were exposed to PMMA or CaSO_4_ beads loaded with either 2.4%, 4.7%, 9.09%, or 13% (w/w) Ga(NO_3_)_3_ in diluted MHB II broth (Figure 3). Control (empty; 0.0%) PMMA and CaSO_4_ beads were not observed to have any antimicrobial activity against either of the two strains tested. In contrast, the Ga(NO_3_)_3_ loaded PMMA beads had antimicrobial activity against both* S. aureus* and* S. epidermidis* over time (Figures 3(a) and 3(b)). Against* S. aureus*, incorporation of Ga(NO_3_)_3_ into PMMA at 2.4%–4.7% (w/w) was associated with a 4- to 6-log reduction during the first three days, whereas no significant antimicrobial activity was observed by day 7. Incorporation of Ga(NO_3_)_3_ into PMMA at concentrations of 9.09%–13% (w/w) had the most dramatic effects reducing bacterial cultures between 6- and 10-logs during the first day, with ≥3-log reductions up to 7 days (Figure 3(a)). Interestingly, for* S. epidermidis*, exposure to Ga(NO_3_)_3_ loaded beads at all of the concentrations tested was observed to have a much greater effect, reducing bacterial cultures, between 4- and 6-log reduction, up to the 7 days evaluated (Figure 3(b)). Similar to the PMMA beads, loading of Ga(NO_3_)_3_ into CaSO_4_ was also observed to have antimicrobial activity against* S. aureus* and* S. epidermidis *(Figures 3(c) and 3(d)). The antimicrobial effect of Ga(NO_3_)_3_ loaded CaSO_4_ was also dependent on the total loaded concentration for* S. aureus, *albeit only the higher concentrations of Ga(NO_3_)_3_ loading, between 9.09 and 13.0% (w/w), were observed to achieve better bacterial reductions, whereas the lower concentrations, 2.4–4.7% (w/w), had less of an effect (Figure 3(c)), which likely reflect the lower levels of Ga(NO_3_)_3_ released at the later time points. In contrast to the effect observed with gallium loaded PMMA, antimicrobial activity against* S. epidermidis* was much more variable, with significant antimicrobial activity observed for beads loaded with ≥4.7% (w/w) (Figure 3(d)).

### 3.4. Effect of Ga(NO_3_)_3_ Loading on Bacterial Colonization of PMMA and CaSO_4_ Beads

**Figure 4:**
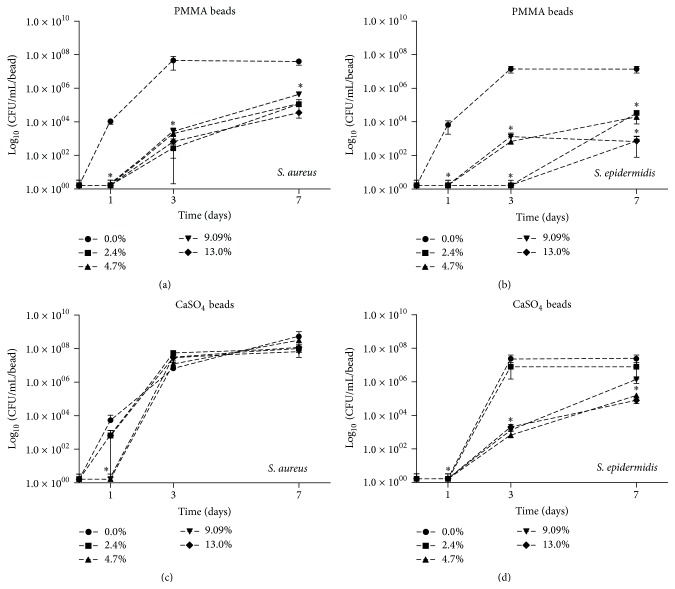
Effect of Ga(NO_3_)_3_ incorporation on bacterial colonization of PMMA and CaSO_4_. Colonization of Ga(NO_3_)_3_ loaded PMMA (a-b) or CaSO_4_ (c-d), 2.4%, 4.7%, 9.09%, and 13.0% (w/w), by* S. aureus *ATCC 12913 and* S. epidermidis *ATCC 12228 over time, as determined by CFU counts (log_10_ CFU/mL per bead). Data is representative of mean ± SD. Statistical analysis was performed using an analysis of variance (ANOVA) with Dunnett's post hoc test; *∗* indicates *p* < 0.05 relative to control.

Due to the associations between biofilm formation and orthopaedic infections, we also evaluated the effectiveness of Ga(NO_3_)_3_ loaded PMMA and CaSO_4_ beads to hinder bacterial surface colonization. The incorporation of Ga(NO_3_)_3_ into PMMA markedly reduced bacteria colonization at days 1 and 3 for* S. aureus* and* S. epidermidis* and up to 7 days for* S. epidermidis* (Figures 4(a) and 4(b)). In contrast, incorporation of Ga(NO_3_)_3_ into CaSO_4_ at even the highest concentrations was only observed to inhibit colonization of* S. aureus* after 1 day, but not thereafter (Figure 4(c)). Loading of Ga(NO_3_)_3_ ≥4.7% (w/w) into CaSO_4_ was observed to significantly reduce colonization of the bead surface by* S. epidermidis* up to the 7 days evaluated (Figure 4(d)).

### 3.5. Activity of Ga(NO_3_)_3_ Loaded PMMA Beads on Established Staphylococcal Biofilms

**Figure 5:**
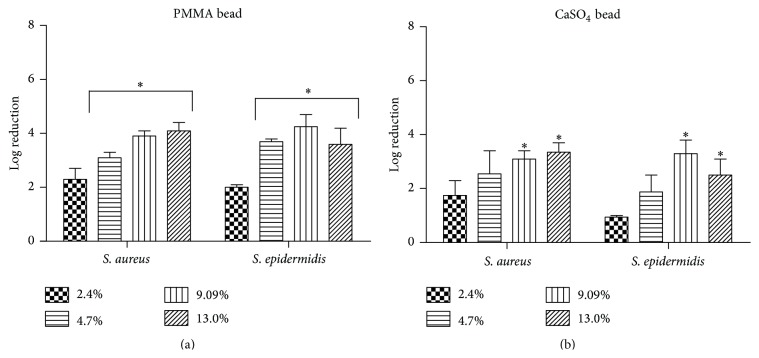
Activity of Ga(NO_3_)_3_ loaded PMMA beads on established biofilms. Effects of Ga(NO_3_)_3_ loaded (a) PMMA and (b) CaSO_4_ beads (% w/w) on established biofilms of* S. aureus *ATCC 12913 and* S. epidermidis *ATCC 12228 following overnight exposure. Data is expressed as the log reductions (mean ± SD) relative to biofilms treated with unloaded control beads. Statistical analysis was performed using an analysis of variance (ANOVA) with Dunnett's post hoc test; *∗* indicates *p* < 0.05 relative to control.

Given the ability of Ga(NO_3_)_3_ loaded beads to reduce bacterial colonization, we assessed whether Ga(NO_3_)_3_ loaded beads also retained activity against established biofilms of* S. aureus* and* S. epidermidis*. Following the 24 h exposure of the preformed biofilms to Ga(NO_3_)_3_ loaded PMMA beads resulted in a 2- to 4-log reduction of viable bacteria within* S. aureus* and* S. epidermidis* biofilms compared to untreated controls, which was highly dependent on the percentage loaded into PMMA (Figure 5(a)). Likewise, CaSO_4_ loaded beads were observed to have a 2- to 3-log reduction of viable bacteria (Figure 5(b)).

## 4. Discussion

Orthopaedic related infections continue to be a significant complication, contributing to the increased overall healthcare associated costs as well as patient associated morbidity [1, 3, 6, 7]. Currently, the guidelines for clinical management of such infections include surgical treatment combined with systemic and/or local antimicrobial therapy. However, the emergence of antimicrobial resistance in addition to the ability of bacteria to develop and persist within biofilms has been shown to limit the effectiveness of this intervention [4, 11, 12, 20], highlighting the need for the development of novel treatment strategies to address this growing clinical challenge. Recently, use of the nonreducible iron analog gallium(III), as the salt Ga(NO_3_)_3_, has been shown to have antimicrobial activity against both Gram positive and Gram negative bacteria* in vitro* and* in vivo* [24–26, 34, 35]. While Ga(NO_3_)_3_ has been approved by the FDA for the treatment of pathological bone loss disorders [28–30], the direct use for treatment of orthopaedic related infections, to our knowledge, has not been evaluated. Therefore, the goal of this study was to assess whether Ga(NO_3_)_3_ could be incorporated into and released from PMMA and CaSO_4_ beads for local delivery into wounds as a potential treatment strategy for orthopaedic related infections.

Given the limitations of this treatment modality, recently there has been a resurgence of efforts to optimize this intervention through use and/or incorporation of unique antimicrobial agents alone or as combinations [4, 41, 42], as well as experimental strategies using various compounds with antimicrobial activities [43–45]. Although these approaches may offer a direct benefit to currently used antimicrobials, the threat of antimicrobial resistance continues to be a major limiting factor; moreover for those experimental strategies the likelihood of their direct clinical use would be limited and not available for some time. Due to the critical role of ferric iron to both normal physiology and virulence for bacteria, the use of Ga(NO_3_)_3_ has been shown to have significant antimicrobial activity against a number of clinically relevant bacteria, including *Staphylococcus* spp.,* P. aeruginosa*, and* A. baumannii* [24–26]. Bone cements, including PMMA and CaSO_4_, are commonly used for local delivery to infected sites to achieve high local concentrations. While these two different carriers offer certain advantages over the other, a major limiting factor is the compatibility of the antimicrobial and/or agent being incorporated [1, 19]. As demonstrated in Figure 2, herein we showed that Ga(NO_3_)_3_ was compatible for incorporation into and release from both PMMA and CaSO_4_. Similar to the release of other previously characterized antibiotics from PMMA [16, 18, 46, 47], release of Ga(NO_3_)_3_ was characterized by a large initial burst, with mean detectable concentrations between 300 and 1500 *μ*M, followed by a sustained release over the seven-day period. For PMMA, incorporation of the lower percentages, 2.4–4.7% (w/w), was observed to have a greater cumulative release (59% and 37%, resp.) compared to those beads loaded with the higher percentages (23–25%). Notably, although Ga(NO_3_)_3_ release was also observed from CaSO_4_, cumulative release was much lower in comparison (21–28%) and not observed to increase with increasing amount of Ga(NO_3_)_3_ incorporated. Although the exact reasons for this are not entirely clear, there is a possibility that CaSO_4_ may have interacted with the gallium and subsequently inhibiting its release. From this assessment of elution kinetics alone, our results indicate that PMMA may be a more suitable carrier for local delivery of Ga(NO_3_)_3_. Of note, while no effects of incorporation of Ga(NO_3_)_3_ on setting time were observed with CaSO_4_ directly, incorporation of increasing concentrations of Ga(NO_3_)_3_ (≥9.09% w/w) into PMMA did extend the curing time significantly (~1 hour). Because antibiotic impregnated beads are prepared during surgical procedures, just prior to placement within wounds, our observations indicate potential limitations to the use of the Ga(NO_3_)_3_ with PMMA, in particular concentrations >9.09%. Importantly, while this is the first study to evaluate the incorporation and release of Ga(NO_3_)_3_ from bone cements, along the lines of our findings are previous reports showing the utility of bone cements, in particular PMMA, for delivery of antimicrobial agents other than antibiotics, including antimicrobial peptides [44] and antiseptics, such as chlorhexidine and quaternary ammonium compounds [45, 48], demonstrating the compatibility of these materials for use with various types of agents, including Ga(NO_3_)_3_ despite limitations.

To be an effective treatment, agents incorporated into PMMA or CaSO_4_ should be appropriate for the organism(s) suspected of causing the infection while also being eluted locally at concentrations sufficient to achieve antimicrobial activity. Consistent with results from the release kinetics studies, the Ga(NO_3_)_3_ loaded PMMA and CaSO_4_ beads were observed to have antimicrobial activity against the planktonic cultures of* S. aureus* and* S. epidermidis, *as demonstrated in Figure 3. Antimicrobial activity of the Ga(NO_3_)_3_ loaded beads was observed to be most effective during the first 3 days, coinciding with the higher elutions of gallium(III) well above the inhibitory concentration against planktonic bacteria, but rapidly losing activity at 7 days, coinciding with release of levels of gallium(III) at levels below this (≤13 *μ*M). In contrast to the antimicrobial activity observed with the gallium loaded PMMA beads, the antimicrobial activities of the gallium loaded CaSO_4_ beads was much more variable demonstrating limited activity against the bacteria tested herein as compared to PMMA. As indicated above, the differences in antimicrobial activity of Ga(NO_3_)_3_ may in part have been explained by interactions of the gallium with CaSO_4_, thereby limiting activity, which would be consistent with the lower cumulative release gallium as well as the reduced antimicrobial activity despite detection of released gallium. Our results demonstrating the activity of Ga(NO_3_)_3_ against staphylococcal species are in line with previous studies [25, 26] and demonstrate the utility of a treatment modality utilizing Ga(NO_3_)_3_ as a treatment strategy for orthopaedic infections.

While antibiotic-loaded bone cements have been shown to be highly effective against planktonic bacteria, use of this treatment modality against biofilms are often limited [4, 18, 49]. This is partly due to the reduced metabolic activity of bacteria within biofilms, limiting the activity of most available antimicrobial agents as the main mechanisms of action target actively dividing cells, and the production of an extracellular polymeric matrix surrounding the community, limiting the diffusion of antibiotics into the biofilm [50, 51]. Because of the association between biofilms and establishment of orthopaedic infections [4] we also evaluated whether the Ga(NO_3_)_3_ loaded beads could limit bacterial colonization (i.e., biofilm formation) of beads; moreover we assessed whether they retained activity against established bacterial biofilms. As demonstrated herein, the Ga(NO_3_)_3_ loaded PMMA beads, and to a lesser extent the gallium loaded CaSO_4_ beads, were observed to reduce bacterial colonization. These findings are particularly important for PMMA, as the beads are nonabsorbable and potentially can become a foreign body that is subject to colonization by bacteria following elution of the incorporated agent [36]. While the ability of Ga(NO_3_)_3_ beads to limit bacterial colonization did diminish over time, our findings are similar to those observed for antibiotic loaded beads, including vancomycin and tobramycin [20]. Importantly, and in contrast to* in vivo* settings, the model used to evaluate surface colonization was performed under highly stringent conditions with daily subjections to bacterial inoculums (~10^6^), which would likely be much greater than that encountered within a wound containing a single infective dose. Future studies evaluating colonization under* in vivo* conditions are warranted given the limitations of our* in vitro* study.

In addition to the antimicrobial activity against planktonic bacteria, Ga(NO_3_)_3_ released from PMMA and CaSO_4_ (Figure 5) was observed to have activity against preformed biofilms of* S. aureus* and* S. epidermidis*. While antimicrobial activity was observed against established biofilms, the effects in comparison to those on planktonic bacteria were markedly reduced, as indicated by the lower log reductions in bacteria, highlighting the issues with treating biofilms. Although the exposure to Ga(NO_3_)_3_ released from PMMA as well CaSO_4_ did not completely eradicate bacteria within the biofilms, our findings demonstrate that Ga(NO_3_)_3_ does retain some activity against biofilms in addition to the planktonic bacteria. This is particularly important because biofilms are thought to play a major role in surgical site infections [52, 53].

While our study provided preliminary evidence indicating the compatibility with bone cements and potential use of Ga(NO_3_)_3_ for treatment of orthopaedic related infections, our current study does have several limitations. First, this study was entirely conducted* in vitro* under ideal conditions that do not accurately recapitulate* in vivo* conditions. As such, to extend the impact of these findings, the results from this study require further evaluation to determine the effectiveness of Ga(NO_3_)_3_ impregnated PMMA and CaSO_4_ beads to reduce microbial burden within an* in vivo* environment. Secondly, although antimicrobial activity following exposure to Ga(NO_3_)_3_ was observed against planktonic, and to a lesser extent against established biofilms of* S. aureus* and* S. epidermidis*, it is important to note that this activity was largely dose-dependent and did not result in sterility. This poses a particular clinical problem, as those organisms remaining following treatment could contribute to relapse of infection within the surgical sites. In light of recent studies demonstrating enhanced activity of conventional antimicrobials in the Ga(NO_3_)_3_, future studies evaluating the use of Ga(NO_3_)_3_ as a combined therapy, rather than a monotherapy, could address this limitation and extend its clinical applications [34]. A third limitation of this study was the fact that we only evaluated incorporation and release from a single type of PMMA and CaSO_4_. As differences in elution kinetics have been observed between the different types of commercially available bone cements [54, 55], it may be relevant to evaluate release of Ga(NO_3_)_3_ from different sources to identify optimal delivery devices for release into surgical sites. Lastly, while Ga(NO_3_)_3_ is currently approved for therapy of cancer-related hypercalcemia, the current therapeutic regimens, based on intravenous infusion, allow for serum levels of 10–20 *μ*M [29, 30]. Based on the studies herein, these levels would be ineffective, requiring levels much higher for achieving antimicrobial activity against* S. aureus* and* S. epidermidis*. While there have been studies extensively evaluating and determining Ga(NO_3_)_3_ to have minimal toxicity* in vitro*, the much higher levels of Ga(NO_3_)_3_ from PMMA and CaSO_4_ well above those levels previously tested warrant further investigation to evaluate biocompatibility and effect on cell function, specifically on osteoblasts and osteoclasts, to determine the limitations of direct application to infected surgical sites.

## 5. Conclusions

The use of antibiotic loaded bone cements is a standard of care used for the prevention and/or treatment of orthopaedic infections. Herein, we show that Ga(NO_3_)_3_ can be loaded into and eluted from PMMA and CaSO_4_ at concentrations effective against both planktonic and biofilms of *Staphylococcus* spp., commonly associated with orthopaedic related infections. Collectively, our* in vitro* findings suggest that local delivery of Ga(NO_3_)_3_ may be an effective strategy for the prevention and/or treatment of orthopaedic related infections. Future studies utilizing animal models are needed to fully characterize the clinical role for this treatment modality.

## Disclaimer

The views expressed herein are the views of the authors and do not reflect the official policy or position of the Department of the Army, Department of the Air Force, Department of Defense, or the US Government. The authors are employees of the US Government and this work was prepared as part of their official duties and, as such, there is no copyright to be transferred.
